# Primary care clinicians’ experiences prescribing HIV pre-exposure prophylaxis at a specialized community health centre in Boston: lessons from early adopters

**DOI:** 10.7448/IAS.19.1.21165

**Published:** 2016-10-11

**Authors:** Douglas S Krakower, Kevin M Maloney, Chris Grasso, Katherine Melbourne, Kenneth H Mayer

**Affiliations:** 1 Division of Infectious Diseases, Beth Israel Deaconess Medical Center, Boston, MA, USA; 2 The Fenway Institute, Fenway Health, Boston, MA, USA; 3 Harvard Medical School, Boston, MA, USA; 4 Gilead Sciences, Foster City, CA, USA

**Keywords:** HIV, pre-exposure prophylaxis, primary care providers, implementation, early adopters, community health centre

## Abstract

**Introduction:**

An estimated 1.2 million Americans have indications for using antiretroviral pre-exposure prophylaxis (PrEP) to prevent HIV acquisition. For many of these at-risk individuals, the best opportunity to learn about and receive PrEP will be during routine visits to their generalist primary care clinicians. However, few generalist clinicians have prescribed PrEP, primarily because of practical concerns about providing PrEP in primary care settings. The experiences of specialized primary care clinicians who have prescribed PrEP can inform the feasibility of PrEP provision by generalists.

**Methods:**

During January to February 2015, 35 primary care clinicians at a community health centre in Boston that specializes in the care of sexual and gender minorities completed anonymous surveys about their experiences and practices with PrEP provision. Responses were analyzed with descriptive statistics.

**Results and discussion:**

Thirty-two clinicians (response rate=91%) completed the surveys. Nearly all clinicians (97%) had prescribed PrEP (median 20 patients, interquartile range 11–33). Most clinicians reported testing and risk-reduction counselling practices concordant with U.S. Centers for Disease Control and Prevention guidelines for PrEP. Clinicians indicated that patients using PrEP experienced medication toxicities infrequently and generally reported high adherence. However, some clinicians’ practices differed from guideline recommendations, and some clinicians observed patients with increased risk behaviours. Most clinicians (79%) rated PrEP provision as easy to accomplish, and 97% considered themselves likely to prescribe PrEP in the future.

**Conclusions:**

In a primary care clinic with specialized expertise in HIV prevention, clinicians perceived that PrEP provision to large numbers of patients was safe, feasible and potentially effective. Efforts to engage generalist primary care clinicians in PrEP provision could facilitate scale-up of this efficacious intervention.

## Introduction

As there are 50,000 new HIV infections in the United States annually, effective HIV prevention strategies are needed [1]. Studies have demonstrated that HIV pre-exposure prophylaxis (PrEP), the use of antiretroviral medications by at-risk uninfected persons, is safe and can reduce HIV transmission among men who have sex with men (MSM) [2], HIV serodiscordant couples [3], men and women with multiple concurrent or sequential partners [4] and persons who inject drugs [5]. In 2012, the FDA approved tenofovir-emtricitabine for use as daily PrEP [6], and the U.S. Centers for Disease Control and Prevention (CDC) issued guidelines recommending PrEP for HIV prevention in 2014 [7].

Generalist primary care providers (PCPs) could play an important role in implementing PrEP, as many of the estimated 1.2 million Americans with indications for using PrEP [8] will receive healthcare from these clinicians. Moreover, there are not enough clinicians with specialized training in HIV-related care to provide access to PrEP for these large numbers of at-risk individuals. Despite CDC guidelines recommending PrEP, however, few PCPs have prescribed PrEP [9]. Surveys of PCPs suggest that they may be hesitant to prescribe PrEP because of multiple concerns about utilizing this intervention, including anticipated patient non-adherence, increased sexual risk-taking and lack of insurance coverage for PrEP [9].

Studies that assess the experiences of early adopter PCPs could provide valuable evidence about the feasibility of prescribing PrEP in primary care. By identifying the challenges faced by early adopters, these studies could also inform programmes designed to engage and train generalist PCPs in PrEP provision. Fenway Health, a community health centre in Boston specializing in healthcare for sexual and gender minorities, is a specialized primary care clinic that has participated in PrEP-related clinical studies since 2006. PCPs at this centre have been prescribing PrEP since 2011, when Fenway Health issued guidelines regarding PrEP provision to its clinicians, and PrEP utilization has since become normative at this centre, where multidisciplinary, team-based PrEP care has been implemented [10]. This study assessed PCP experiences and practices with PrEP provision at Fenway Health to gain a greater understanding of early adopter clinicians’ perspectives on implementing PrEP in primary care.

## Methods

During January to February 2015, all PCPs (*n*=35) at Fenway Health were invited to complete anonymous 35-item surveys assessing experiences with PrEP provision. Surveys assessed provider demographics, practice characteristics, experiences and practices with PrEP provision, perceptions about feasibility and future prescribing intentions. Providers were asked to indicate their perceptions of patients’ experiences with several aspects of PrEP utilization, including: *Financial barriers* (“Which of the following barriers has prevented a patient from taking PrEP? Lack of insurance coverage for the cost of PrEP medications; Patient unable to pay out-of-pocket costs; None of the above”); *Medication adherence* (“In general, how are levels of adherence to PrEP among your patients? Very poor, Poor, Fair, Good, Very good, Excellent”); *Impact of medication intolerance on adherence* (“In general, how have side effects from PrEP negatively impacted patient adherence? Not at all, A small degree, A moderate degree, A great degree”); and *Risk compensation* (“Have your patients reported any behaviour changes while using PrEP?” followed by questions about changes in the frequency of condom use during sex, numbers of sexual partners and the frequency of having sex with HIV-positive persons: Less often/A smaller number, No change, More often/A greater number, Have not specifically assessed this behaviour). Respondents were also asked about changes in rates of sexually transmitted infections (STIs) among patients using PrEP (“Have your patients experienced more STIs as a result of using PrEP? No patients, Some patients, Many patients, Have not specifically discussed/tested for this.”) Questionnaire development included review by experts in HIV prevention for content validity and cognitive testing with clinicians for face validity and comprehension. After providing consent, clinicians completed self-administered online surveys. Participants were compensated $25. Responses were characterized with descriptive statistics (SAS v.9.4). The Institutional Review Board at Fenway Health approved all study procedures and participants provided consent prior to engaging in study activities.

## Results and discussion

### Sample characteristics

Thirty-two clinicians completed surveys (response rate=91%). Participants’ median age was 37 years (interquartile range (IQR) 34–51) and 53% were female; 81% identified as White, 13% as Asian, 3% as Hispanic/Latino and 6% as Multiracial. Fifty per cent of respondents self-identified as gay. The sample was professionally diverse and included physicians (59%), nurse practitioners (22%) and physician assistants (19%). Most participants provided primary care to HIV-positive patients (median 50 patients, IQR 15–130).

### Prescribing experiences and beliefs

All respondents (*N*=32) believed PrEP to be highly efficacious. Nearly all participants (97%) had prescribed PrEP to at least one patient. Thirty-one respondents had prescribed PrEP to a median of 20 patients from diverse populations, including MSM and members of HIV serodiscordant couples, among others (Table 1).

**Table 1:** Primary care providers’ experiences with prescribing HIV pre-exposure prophylaxis at a specialized community health centre in Boston

	*N* (%) (*n*=31)
Characteristics of patients receiving PrEP	
Men who have sex with men	31 (100)
HIV-serodiscordant couples	26 (84)
People with a sexually transmitted infection	24 (77)
People who change sex partners frequently	24 (77)
Persons who have used post-exposure prophylaxis	23 (74)
People who exchange sex for money, drugs or other goods	12 (39)
People who inject drugs	10 (32)
Financial barriers to initiating PrEP	
Patient lack of insurance coverage	15 (48)
Patient unable to pay out-of-pocket costs	14 (45)
Neither of the above	12 (39)
Perceptions of patient adherence	
Excellent	6 (19)
Very good	17 (55)
Good	8 (26)
Fair	0
Poor	0
Impact of medication intolerance on patient adherence	
Not at all	13 (42)
A small degree	18 (58)
A moderate degree	0
A great degree	0
Medication discontinuation	
Has discontinued PrEP for ≥1 patient	20 (65)
Reasons for discontinuation	
Patient preference	18 (58)
Patient-reported intolerance of medication	6 (19)
Patient did not adhere to PrEP medication	6 (19)
Patient did not attend monitoring or counselling visits	5 (16)
Medication toxicities discovered on lab testing	4 (13)
Lack of insurance coverage	3 (10)
Prohibitive out-of-pocket expenses	3 (10)
HIV acquisition	1 (3)
Increased HIV risk behaviours while using PrEP	0
Use of PrEP other than as directed	0
Risk compensation	
Condom use during anal sex	
Less often	13 (42)
No change	16 (52)
More often	2 (6)
Did not assess	0
Number of sexual partners	
A smaller number	0
No change	23 (74)
A greater number	7 (23)
Did not assess	1 (3)
Having sex with HIV-positive persons	
Less often	0
No change	21 (68)
More often	9 (29)
Did not assess	1 (3)
More frequent sexually transmitted infections	
No patients	19 (61)
Some patients	10 (32)
Many patients	0
Did not assess	1 (3)
Feasibility and future prescribing intentions	
Perceived difficulty of prescribing PrEP	
Very easy	11 (35)
Somewhat easy	13 (42)
Neither easy nor challenging	5 (16)
Somewhat challenging	2 (6)
Very challenging	0
Likelihood of future prescribing	
Very likely	24 (77)
Likely	6 (19)
Unlikely	0
Very unlikely	1 (3)

Responses are restricted to the 31 respondents who had prescribed PrEP. Percentages exceed 100% for responses that asked participants to select all that apply.

### Financial barriers

Nearly half of the respondents indicated that lack of insurance or co-pays had prevented patients from using PrEP (Table 1).

### Testing and counselling

**Figure 1:**
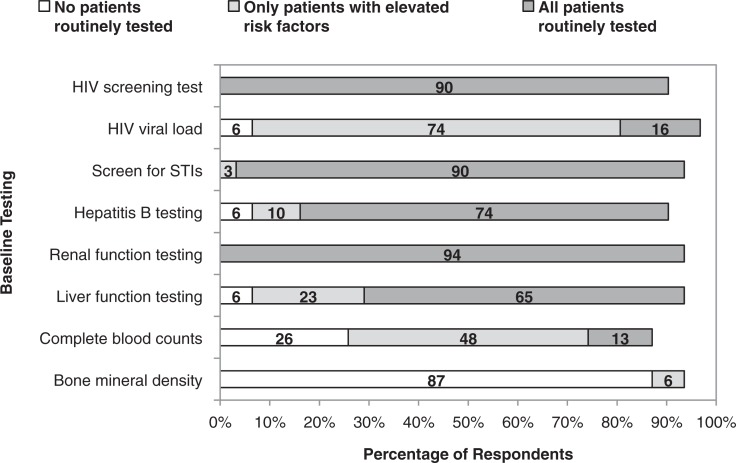
**Primary care providers’ reported testing practices before prescribing HIV pre-exposure prophylaxis.** Primary care providers (*n*=31) indicated whether they performed specific tests for no patients, only patients they perceived to be at elevated risk for complications or all patients prior to prescribing PrEP. Guidelines recommend testing for renal function, HIV, STIs and hepatitis B prior to PrEP initiation; guidelines do not recommend liver function tests, complete blood counts or bone mineral density tests. Numbers inside bars represent percentage of study participants indicating each response category; numbers inside bars do not total to 100% because of missing responses to individual questions.

Most participants performed testing for renal function, HIV and STIs for all patients initiating PrEP, in accordance with CDC guidelines (Figure 1) [7]. Respondents generally only utilized HIV viral load testing for patients with elevated risk for having acute HIV infection, including patients with recent high-risk exposures to HIV or symptoms consistent with acute HIV. For patients using PrEP, most providers conducted quarterly risk-reduction counselling (71%), HIV testing (77%) and screening for asymptomatic STIs (68%); the remaining providers did so at four to six month intervals except for one provider who screened for STIs annually.

### Adherence and discontinuation

Patient adherence to PrEP was perceived to be high (Table 1). Providers generally thought that side effects from PrEP did not commonly affect adherence. Two-thirds of respondents had ≥1 patient discontinue PrEP, for various reasons (Table 1), including HIV acquisition (one respondent).

### Risk compensation

Some PCPs indicated that patients had disclosed increased sexual risk behaviours after initiating PrEP, including using condoms less often during anal sex (42%), having more sexual partners (23%) and having sex with HIV-positive persons more frequently (29%) (Table 1). One-third of respondents perceived that some of their patients had experienced increased frequencies of STIs after initiating PrEP.

### Feasibility and prescribing intentions

A majority of respondents described PrEP provision as “Very easy” (35%) or “Somewhat easy” (42%), and nearly all clinicians considered themselves likely to prescribe PrEP in the future (Table 1).

## Discussion

This is one of the first studies to demonstrate provision of PrEP by multiple primary care clinicians to large numbers of patients at a community health centre. Clinicians reported prescribing practices that were largely consistent with CDC guidelines [7], and they perceived that patients were generally adherent to PrEP and infrequently needed to discontinue PrEP due to adverse effects. The most common reason for discontinuing PrEP was patient preference, which is consistent with prior studies [11] and may be because patients prefer not to use PrEP during periods when they perceive themselves to be at lower risk for HIV acquisition. Overall, the results suggest that clinicians perceived that PrEP was used by their patients in a safe and potentially effective manner during primary care. These findings are consistent with studies demonstrating the feasibility of PrEP provision in other clinical settings [12,13]. The major implication of these findings is that PrEP provision by generalist primary care clinicians, which will be essential for scaling-up PrEP nationally, might also be feasible if generalists are provided with training on how to prescribe PrEP.

Clinicians reported challenges with providing PrEP. Half of the respondents indicated that financial barriers had prevented patients from utilizing PrEP, similar to prior studies in which clinicians have cited multiple insurance-related barriers to PrEP provision [14]. Thus, providers and patients should be educated about medication assistance programmes from the manufacturer of tenofovir-emtricitabine (www.truvada.com/truvada-patient-assistance), as out-of-pocket costs for PrEP care can amount to several thousand US dollars annually for some patients with healthcare insurance [15]. Some clinicians did not perform all testing (e.g. hepatitis B testing) or risk-reduction counselling in accordance with CDC guidelines [7], and some performed tests that are not recommended (e.g. liver function testing). Studies to understand the reasons for deviations from recommended practices could guide efforts to improve practices and optimize guideline recommendations. Finally, a minority of clinicians observed increased sexual risk-taking with PrEP use, consistent with other studies [13]. As PrEP is highly effective when taken daily [2–5,7,16], adherent patients who increased sexual risk-taking while using PrEP likely remained at lower risk for acquiring HIV than if they had not used PrEP. This suggests that clinicians should not withhold PrEP from patients because of concerns about risk compensation. However, increased risk behaviours could promote transmission of other STIs, which have been detected at high rates among persons using PrEP in care settings [13,16]; so clinicians who prescribe PrEP will need to be rigorous about screening for and treating STIs.

This study has limitations. Recruitment was limited to a single community health centre, so the study findings may not be generalizable to clinicians who practice at other centres. Respondents had experience with prescribing antiretroviral medications for HIV treatment, and they practiced at a health centre that conducted prior PrEP research. Therefore, these clinicians may have been more knowledgeable about PrEP and more receptive to adopting PrEP into practice than less experienced PCPs. Generalist PCPs may be less likely than participants in this study to conduct sexual health assessments routinely during primary care, which could impede PrEP provision in general primary care settings. Participants may have reported practices consistent with guidelines because of social desirability bias despite the anonymous survey, so objective assessments of clinicians’ practices (e.g. reviews of medical records) would strengthen confidence in our findings. Similarly, clinicians’ perceptions of their patients’ behaviours, such as those related to adherence and sexual risk behaviours, could be inaccurate as a result of recall bias or incomplete disclosure of risk behaviours by patients, so direct assessments of patient behaviours are needed. A strength of the study is the unusually high response rate for surveys of clinicians (91%) [17], which suggests that non-response bias is likely to be limited despite the small sample size.

## Conclusions

In this study, clinicians with specialized expertise in HIV prevention perceived that PrEP provision to large numbers of patients was feasible, safe and potentially effective in a primary care clinic. As most persons at risk for acquiring HIV will not have access to clinics with specialized knowledge of HIV prevention, programmes to train more generalist primary care clinicians to prescribe PrEP should be developed as a way to ensure wider and more equitable access to PrEP nationally.
